# Characterization of Ex Vivo Expanded Tumor Infiltrating Lymphocytes from Patients with Malignant Melanoma for Clinical Application

**DOI:** 10.1155/2011/574695

**Published:** 2011-06-18

**Authors:** Niels Junker, Per thor Straten, Mads Hald Andersen, Inge Marie Svane

**Affiliations:** ^1^Center for Cancer Immune Therapy (CCIT), Department of Oncology, University Hospital Herlev, Herlev Ringvej 75, 2730 Herlev, Denmark; ^2^Center for Cancer Immune Therapy (CCIT), Department of Hematology, University Hospital Herlev, Herlev Ringvej 75, 2730 Herlev, Denmark

## Abstract

Clinical trials of adoptive transfer of autologous tumor infiltrating lymphocytes (TILs) to patients with advanced malignant melanoma have shown remarkable results with objective clinical responses in 50% of the treated patients. In order to initiate a clinical trial in melanoma, we have established a method for expanding TILs to clinical relevant quantities in two steps with in 8 weeks. Further characterization of expanded TILs revealed an oligoclonal composition of T-cells with an effector memory like phenotype. When autologous tumor was available, TILs showed specific activity in all patients tested. TIL cultures contained specificity towards tumor cells as well as peptides derived from tumor-associated antigens (TAAs) during expansion procedures.

## 1. Introduction

The incidence of malignant melanoma is increasing worldwide, and upon dissemination has a very poor prognosis [1]. Only two systemic treatments are approved for disseminated disease and encompass IL-2 based immunotherapy (16% response rate and 6% complete responses) [2] and dacarbazine (6%–15% response rate with no improved survival) [3]. However, results from clinical trials of TIL-based immunotherapy conducted at two centres has shown 50% response rates in patients with advanced disease, and responses were long lasting [4, 5]. TILs were reported to be dominated by CD8^+^ T-cells and mediate specific killing of autologous tumor in most patients [6]. Information on TAA-derived peptide specificities in TIL has mainly shown the occasional large frequency of MART-1 and gp100 specific T-cell populations. On the other hand, results on the clonotypic and phenotypic composition has been scarce; one publication has revealed a mixed clonal content of TIL by FACS analysis [7], and two recent studies report surface markers identical to memory like effector T-cells from a limited patient material [8, 9]. 

In our study, we have analysed TIL characteristics from 17 melanoma patients, whereof five have undergone lymphodepletion and TIL-based ACT with low-dose IL-2.

## 2. Materials and Methods

### 2.1. Patients

Patients referred to surgery for primary or recurrent stage III-IV malignant melanoma were eligible for the study. The study protocol was approved by the local ethics committee, and all patients were included after signing informed consent. Tumor material from the patients was obtained from the surgically removed tumour within 30 minutes after surgery.

### 2.2. TIL Bulk Cultures and Rapid Expansion

The TIL culturing method was adapted from Dudley et al. [10] constituting a two-step expansion process: (I) initiating bulk cultures and (II) rapid expansion of selected bulk cultures with a proliferative potential. Following surgical removal of tumor tissue from patients with MM the tumour sample were cut into 1-2 mm fragments. Fragments were subsequently placed individually in 24-well culture plates (Nunc, Denmark) and maintained in 2 mL of culture medium (CM) containing RPMI1640 (Invitrogen), penicillin, streptomycin, fungizone (Bristol-Myers Squibb), 10% human serum (Sigma) and 7300 or 6000 IU/mL IL-2 (Aldesleukin, Novartis). Each fragment initiated an individual TIL culture which was maintained separately during subsequent expansion and activation. 

Bulk cultures were selected for further expansion according to a rapid expansion protocol (REP). TIL were cocultured with irradiated (40 Gy) allogeneic PBMCs serving as feeder cells in a ratio of 1 : 200 in a 1 : 1 mixture of CM and AIM-V (Invitrogen) initially with 10% HS, and containing 30 ng/mL OKT-3 (Cilag AG, Suisse) and 7300 or 6000 IU/mL IL-2 (Aldesleukin, Novartis) in upright T-flasks. REPs for preclinical purposes generally were initiated from 1 × 10^5^ TIL per flask, while 1 × 10^6^ TIL were used per flask in clinical scale REPs. On day 5, half of the medium was replaced with fresh medium containing AIM-V, CM with 10% HS and 7300 IU/mL IL-2. From there on, the TIL concentration were maintained at 1 × 10^6^ cells/mL by adding AIM-V supplemented with Fungizone and 7300 or 6000 IU/mL IL-2. Half of the patients TIL where cultured in 7300 IU/ml IL-2, while the other half received 6000 IU/mL IL-2 during culturing.

### 2.3. Viability

Cell counting and viability testing were performed by microscopy. Cells were stained with trypan blue followed by counting of live and dead cells in a haemocytometer.

### 2.4. Sterility Tests

Bulk and REP cultures were intermittently sampled for microbiological testing of fungal and bacterial contamination.

### 2.5. Peptides

We used the following HLA-A2 restricted peptides: SUR1M2 (LMLGEFLKL), HTERT P540 (ILAKFLHWL), Cyclin B1 204 (ILIDWLVQV), MART-1 27–35 (AAGIGILTV ), and NY-ESO 1 157–165 (SLLMWITQC).

### 2.6. Cell Lines

Autologous tumor cell lines were established from tumor fragments by outgrowth in 24 well or 6 well plates (Nunc) in medium consisting of RPMI1640 (Invitrogen), penicillin, streptomycin, fungizone, 10% fetal calf serum (Invitrogen), and SoluCortef (Pfizer). 

Tumor cells were cryopreserved in 90% FCS and 10% DMSO (Hospital Pharmacy, RegionH, Copenhagen, Denmark) and stored at −140°C.

### 2.7. Flow Cytometry

Phenotyping were conducted using a FACS-Aria with Diva software (from BD) and fluorescence conjugated monoclonal antibodies (mAb) against CD3 APC-Cy7, CD4 APC, CD8 PerCP, CD25 PE, CD27 PE, CD45RA FITC, CD45RO PE, CD56 PE (all from BD), CCR7 FITC (R&D systems), CD16 FITC (Dako), CD28 FITC (Immunotech), CD62Ligand PE (BD Pharmingen), and CD57 FITC (BD Pharmingen) along with corresponding isotypes.

### 2.8. T-Cell Receptor (TCR) Clonotype Mapping by Denaturing Gradient Gel Electrophoresis (DGGE)

RNA was extracted using the NucleoSpin RNA II (Macherey-Nagel, Germany). cDNA synthesis and quantitation of cDNA in each sample was carried out as previously described [11]. 

For TCR clonotype mapping, cDNA was amplified using a primer panel covering the 24 BV region families of the TCR. Resulting PCR products are suited for DGGE [12, 13]. Amplifications were carried out in a total volume of 45 *μ*L containing 1xPCR buffer (50 mM KCl, 20 mM Tris pH 8.4, 2.0 mM MgCl_2_, 0.2 mM cresol red, 12% sucrose, 0.005% (wt/v) BSA (Boehringer-Mannheim, Mannheim, Germany)), 2.5 pmol of each primer, 40 mM dNTPs (Pharmacia LKB, Uppsala, Sweden) and 1.25 units of AmpliTaq polymerase (Perkin Elmer Cetus Corporation, Emeryville, Calif, USA). Parameters and conditions used for amplification were 94°C for 30 sec., 60°C for 60 sec., and 72°C for 60 sec., as described, in [11, 12]. 

For DGGE 10 *μ*L aliquots were loaded onto a denaturing gradient gel containing 6% polyacrylamide and a gradient of urea and formamide ranging from 20% to 80%. Gels were run at 160 V for 4.5 h in 1x TAE buffer kept at a constant temperature of 56°C. After electrophoresis, the gel was stained with SYBR Green I (Molecular Probes, Oregon, USA) and visualized using the FLA-3000 fluorescence detection system (FUJI film, Science Imaging Scandinavia, Sweden).

### 2.9. Elispot INF*γ* Measurement

Antitumor activity was assessed with Elispot INF*γ* quantification as described previously [14]. In brief, nitrocellulose bottomed 96 well plates (Multiscreen MAIP N45; Millipore, Denmark) were coated with INF*γ* capturing antibody (1-DIK; Mabtech, Sweden) and further washed and blocked with RPMI 1640. A maximum of 1 × 10^5^ effector cells per well were either added alone when stimulated by peptides, or in coculture with target cells (1 × 10^4^ cells per well) consisting of autologous tumor cells. After a four-hour or overnight incubation period, the medium was discarded and wells washed followed by application of secondary biotinylated antibody (7-B6-1-Biotin; Mabtech). The plates were incubated for one hour, further washed, and avidin-enzyme (Streptavidin; Mabtech) conjugate, were added to each well followed by one-hour incubation at room temperature. Succeedingly, the wells were washed and the enzyme substrate NBT/BCIP (nitro blue tetrazolium/5-bromo-4-chloro-3-indolyl phosphate; Mabtech) were added into each well. The plates incubated at room temperature for 2 to 10 minutes, while emerging purple spots developed. The reaction was terminated with tap water. Spots were counted with the ImmunoSpot Series 2.0 Analyzer (CTL Analyzers) and the frequency of tumor specific TIL could be calculated from the numbers of spot forming cells. The assays were preferably done in triplets or in duplicates in case of low cell numbers.

### 2.10. Cr Release Assay

A standard Cr^51^-release assay was used to quantify the specific cytotoxic ability of selected TIL cultures. In brief, 5 × 10^3^ Cr^51^-labeled tumor cells (duplicates or triplicates) were cocultured with TIL (maximum E : T ratio of 100 : 1 and titrated) in RPMI containing 10% FCS for a 4 hour incubation period. Thereafter, Cr^51^-release was measured and percentage of tumor lysis calculated as (#count − Min count)/(Max count −Min count) × 100%.

### 2.11. Statistical Analysis

We utilized Graphpad Prism statistical software to analyse for statistical differences, using a paired two-tailed *t *test. *P* values <.05 were considered significant.

## 3. Results

### 3.1. Patients

Tumor material were obtained from 17 patients with either locally advanced or advanced disease from metastasis localized either in lymph nodes (majority of specimens) or subcutaneously. A minimum requirement of 1 cm^3^ of tumor was needed to ensure sufficient material for TIL expansion. The mean age was 62 years with an equal gender distribution. 12 of the patients had only been treated surgically prior to inclusion, while five patients who were included in our recent established clinical pilot trial had previously received IL-2 and/or DC vaccination based immunotherapies. Patients showed the following distribution of HLA-A types: one HLA-A1+, two HLA-A1/A3+, one HLA-A3+, two HLA-A3/A11+, one HLA-A11+, one HLA-A3+/A2+, four HLA-A2+, one HLA-A2/24+, and four non-HLA-A1/A2/A3/A11/A24.

### 3.2. TIL Expansion Kinetics

Lymphocytes migrated out from the fragments within two-to-five days and expanded into a confluent layer before splitting the wells. Each well initiated an individual bulk culture and were kept separated from other cultures. TIL bulk cultures expanded to at least 5 × 10^7^ cells were considered sufficiently expanding. This was obtained in 15 out of 17 patients (88%) in 6% to 100% of the bulk cultures (mean 58%) within 3–5 weeks. We found that growth rates varied markedly even between cultures from the same patient, and there was no difference in success rate of TIL growth from LN or SC tumor material, nor between the IL-2 concentrations (data not shown). 

**Figure 1:**
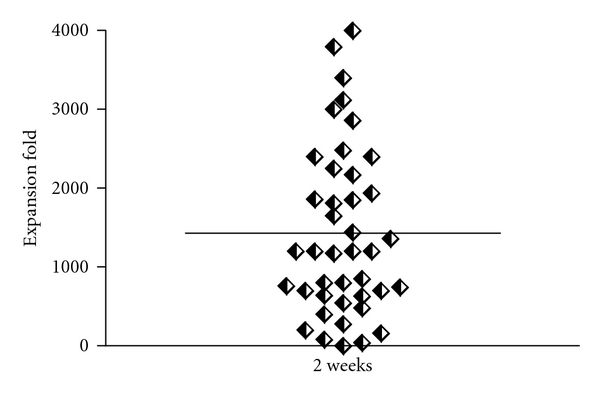
REP fold-expansion. During two weeks 41 TIL cultures (represented by single diamonds) reached a mean 1400 expansion fold.

We next tested the proliferative potential of a range of bulk cultures from 12 of the 15 patients with sufficiently growing TIL. This rapid expansion procedure (REP) involves the addition of allogeneic feeder cells and a CD3 antibody and has shown to increase TIL expansion rates considerably, in previously reports in both melanoma and head and neck squamous cell carcinoma. Again, the kinetics could vary between cultures from the same patient; however, the procedure could efficiently expand TIL bulk cultures to over a 1000-fold in more than half of the cultures in 2 weeks (Figure 1). 

### 3.3. Phenotypes and Clonal Composition

TIL were visualized in the microscope, showing a blasted morphology related to actively dividing lymphocytes laying either as single cells or in clusters/clones. 

**Figure 2:**
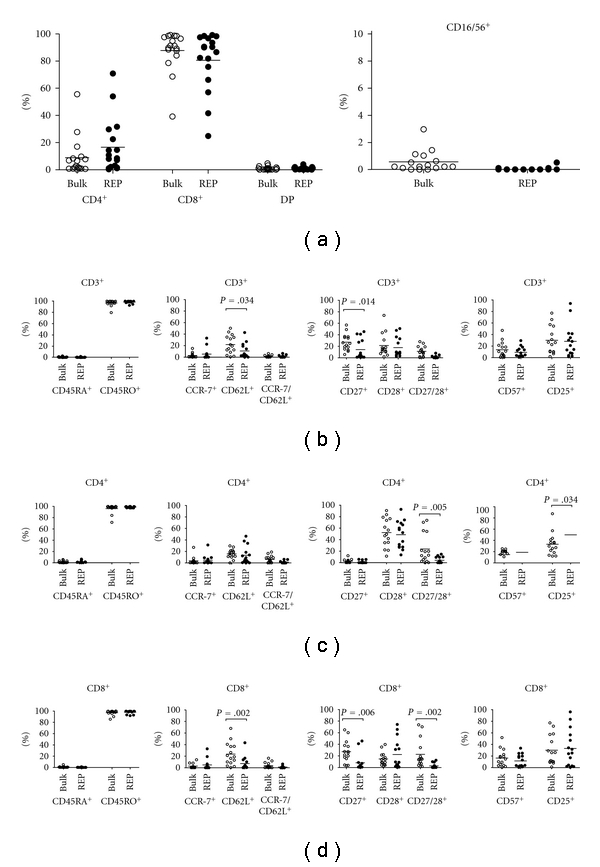
Phenotypes. FACS determination of phenotypes of TIL cultures pre- (open circles) and post-REP (closed circles) are represented from six patients. The overall T-cell effector memory like phenotype (CD3^+^CD45R0^+^CCR-7^ Lo ^) is preserved after REP with a sustained low expression of CD57 and intermediate CD25 expression. CD28 remains unchanged, while CD62L and CD27 is downregulated, indicating a differentiation towards a later effector stage.

In acquisition of cells by flow cytometry, gating of viable cells was performed on the basis of the forward and side scatter dot plots. T-cells (CD3^+^) predominated the cultures, while NK cells (CD16/56^+^) (Figure 2) were consistently absent. In bulk cultures, we observed a heterogeneous CD4^+^ and CD8^+^ T-cell distribution among cultures inter- and intraindividually. There was, however, an overall skewing towards a CD8^+^ (mean = 74%  ± 24%, range 30%–94%) T-cell predominance in relation to CD4^+^ T-cells (mean = 19.5%  ± 23.5%, range 1%–64%). Next, we investigated the occurrence of surface markers identifying T-cell memory subsets, or alternatively, a differentiation path of effector cells, in the overall CD3^+^ population, and among CD4^+^ and CD8^+^ T-cell subsets in comparison to TIL after two weeks of REP (Figure 2). Overall, there was a distinct predominance of CD45RO^+^ and CCR-7^ Lo/−^ T-cell populations before and after REP identifying the cells as T effector memory like. 

TIL were further characterized by surface markers according to a proposed model of effector CD8^+^ T-cell differentiation stages by Gattinoni et al. [15]. Expression of the lymphoid homing marker CD62L was significantly reduced after REP in the CD3^+^ population and the CD8^+^ subsets and remained unchanged among the CD4^+^ subsets. Concerning expression of costimulatory markers, we observed a significantly higher expression of CD27 among CD8^+^ bulk TIL compared to CD4^+^ cells, while CD4^+^ cells had sustained higher CD28 expression in bulk cultures and after REP. Although there was a relatively high percentage of CD27/28 double positive cells in a few bulk TIL, they were downregulated after REP. Finally, there was a significant increase in the high-affinity IL-2 receptor (CD25) after REP in the CD4^+^ population. In conclusion, the CD8^+^ population express surface markers (CD45RO^+^, CCR-7^ Lo ^, CD62L^ Lo ^, CD27^ Lo ^, CD28^ Lo ^, and CD57^ Lo ^) resembling intermediate to late-stage effector cells as reported by other groups [8, 9, 16]. 

Selected expanded cultures were analyzed for the presence of clonally expanded T cells by RT-PCR/DGGE-based TCR clonotype mapping. Analysis revealed the presence of at least 10 different T-cell clonotypes in bulk cultures as well as in rapidly expanded cultures (data not shown). The results support our previous findings in expanded TIL from head and neck cancer patients, that expansion by high-dose IL-2 and CD3 antibody seems to support the continued expansion of bulk T-cell clones.

### 3.4. Sustained Functional Capacity during Expansion

**Figure 3:**
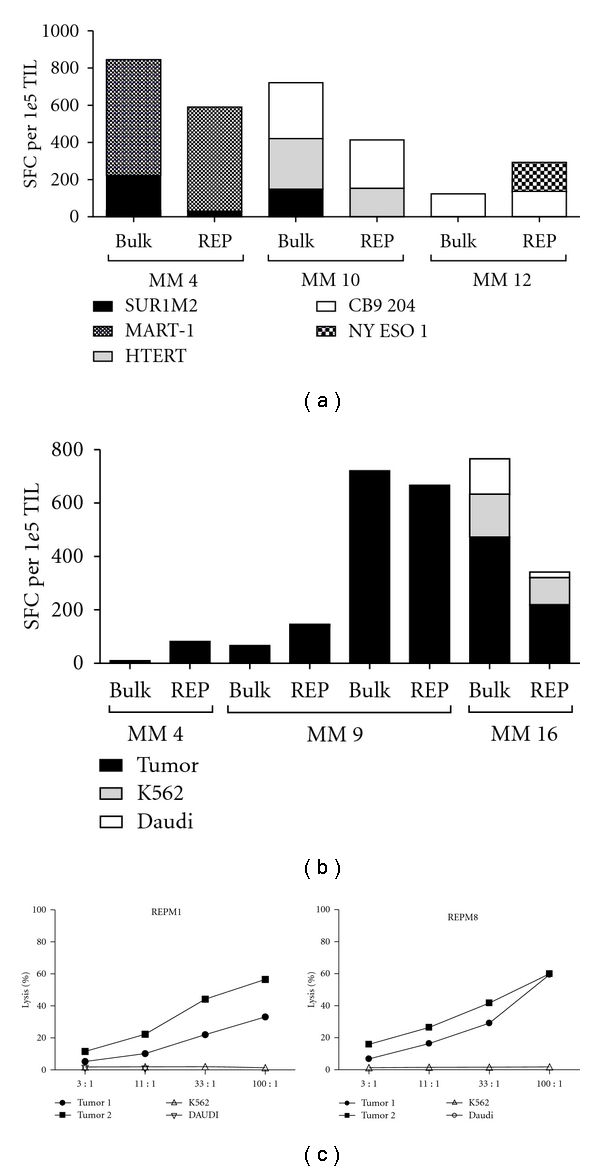
Functional capacity. Determination of TAA peptide-specific populations in TIL pre- and post-REP. Results from Elispot detection of INF*γ* in three patients exemplifies the general tendency of specificity retention, decline, and/or increase as a consequence of unspecific stimulation during expansion procedures, (a) Autologous tumor cell lines were available from four patients, and all showed TIL with antitumor activity by measuring INF*γ* in Elispot. Representative results of TIL from three patients show retained tumor-specific activity. However, in patient MM + 16 we found a component of unspecific NK/LAK cell activity, which seemed to decline after REP, (b). Example of preserved lytic capacity of TIL from MM 11 after REP. Both cultures show specific killing of two established autologous tumor cell lines. Interestingly, Tumor 1 seems more immunogenic than Tumor 2, (c).

TIL cultures from eight patients were selected to scrutinize the presence of specific T-cell populations in bulk cultures and after REP. Peptides derived from over expressed (Telomerase, Survivin, and Cyclin B1), differentiation (MART-1) and cancer testis antigens (NY-ESO-1) served as known targets, while autologous tumor cell lines presented a panel of unknown antigen specificities. Elispot detection of INF*γ* release upon antigenic stimulation revealed a sustained functionality of TIL after REP. Due to the high sensitivity of the assay, we could follow the presence and loss of low-frequency single-peptide-specific T-cell populations (Figure 3(a), occurring as a consequence of an increase or decrease in cell number of a given specific cell population, during the unspecific expansion procedures provided by IL-2 and anti-CD3. Autologous tumor cell lines were available in four patients, and all patients contained TIL showing antitumor activity in Elispot (Figure 3(b) and data not shown). The presence of autologous tumor-specific T-cell populations was more resistant during REP and showed a sustained functional capacity. Although T-cell-specific antitumor activity was predominating in TIL, we observed LAK/NK cell activity in a few cultures (Figure 3(b)) by unspecific engaging the cell lines K562 and Daudi. Finally, we confirmed a sustained tumoricidal capacity of TIL after REP (Figure 3(c)) indicating that TIL expanded to clinical relevant numbers (2400- and 4000-fold) can engage and kill autologous tumor.

## 4. Conclusion

We were able to establish sufficiently expanding TIL bulk cultures in five weeks from the majority of included melanoma patients. Further expansion by REP generated a mean expansion fold of 1400 in two weeks, ensuring the feasibility to reach clinical relevant quantities for clinical testing. Based on earlier studies of T-cell therapy of melanoma patients were as low as 1,3 × 10^9^ infused cells containing 30% MART-1-specific CD8^+^ T-cells mediated a complete clinical response [17], we estimate that a minimum of 3 × 10^9^ cells are required to obtain a therapeutic effect. Cell-based analysis revealed an oligoclonal composition of T effector memory cells, predominated by CD8^+^ cells showing an intermediate to late stage of differentiation after REP. TIL retained the functional capacity measured by INF*γ* release and lytic activity against autologous tumor. Notably, we did not find differences between the two doses of IL-2 used during TIL culturing, and even further lowering of IL-2 dose to 3000 IU/mL is now the standard used in TIL expansion at other centres. Finally, there were no significant influence on TIL expansion kinetics or phenotypes by pretreatment, age or performance status of the patients.

## 5. Perspectives

In a recently initiated clinical trial of TIL-based ACT, low-dose IL-2, and lymphodepletion preconditioning, one out of five treated melanoma patients has obtained an ongoing partial response (+13 months). We are currently screening the TIL cultures for the occurrence of tumor associated antigen (TAA) specificities by measuring INF*γ* in Elispot. This enables us to identify the specific combination of TAA specificities in each patient, which potentially can be identified during immune monitoring of the patient samples. In addition, we are establishing and validating a flow cytometry-based method of identifying TAA-specific T-cell populations and obtain information on the kinetics of T-cell memory and effector stages before and after treatment. Information providing more insight into the prognostic values of adoptively transferred TIL.
